# Misdiagnosis of placental mesenchymal dysplasia as pregnancy with hydatidiform mole: A case report and literature review

**DOI:** 10.1097/MD.0000000000033438

**Published:** 2023-04-14

**Authors:** Ping Tang, Xiaoying Jin, Jiarui Li, Liyan Zhang, Yuan Li, Shengfeng Xu

**Affiliations:** a Jiaxing Maternity and Children Health Care Hospital/TheAffiliated Women and Children’s Hospital of Jiaxing University, Jiaxing, Zhejiang, China; b Department of Orthopaedic Surgery, The First Affiliated Hospital, Zhejiang University School of Medicine, Hangzhou, China.

**Keywords:** case report, hydatidiform mole, misdiagnosis, placental mesenchymal dysplasia

## Abstract

Placental mesenchymal dysplasia (PMD) is a rare placental disease frequently associated with severe maternal and/or fetal complications. Its sonographic appearance is very similar to that of a hydatidiform mole. Hence, PMD is easily misdiagnosed as a hydatidiform mole. In this study, we reported the clinical features of PMD and analyzed its relationship to other severe maternal and/or fetal complications.

**Patient concerns:**

A 28-year-old female, gravida 2, para 1, was referred to our maternal and child health hospital at 15 weeks + 2 days due to an ultrasonic diagnosis of partial hydatidiform mole. Analysis of chromosome karyotype + mononucleotide-based gene microarray by amniocentesis at the 19th week of gestation showed that fetal amniocentesis chromosome 46, XN, high-resolution chromosome microarray analysis of Affymetrix CytoScan 750K Array revealed a 210 kb fragment deletion in chromosome 2p16.3 containing NRXN1, an OMIM gene, the deleted fragment was derived from a mother with a normal phenotype. The pregnant woman delivered a healthy baby girl at 36 weeks + 5 days.

**Diagnoses:**

Based on the clinical characteristics, imaging, and genetic test findings, the postoperative diagnosis was PMD.

**Intervention:**

Because of “Scar uterus” and “Pregnancy with hydatidiform mole,” a 2490 g female infant was delivered by cesarean section at 36 weeks + 5 days of gestation with an Apgar score of 9/9.

**Outcomes:**

The maternal human chorionic gonadotropin level decreased to the normal range after 10 days of delivery, and the infant was not found abnormal after 3 months of follow-up.

**Lessons:**

From our cases and 19 other cases obtained from the PMD literature review are associated with unique clinical, laboratory, and imaging features compared with a hydatidiform mole, such as stained glass sign, normal serum levels of serum human chorionic gonadotropin, elevated alpha-fetoprotein levels and female fetus.

## 1. Introduction

Placental mesenchymal dysplasia (PMD) is a rare placental disease characterized by placental enlargement and grape-like blisters, often associated with severe maternal and/or fetal complications. PMD is characterized by varying expressions of placentomegaly, aneurysmally dilated chorionic plate vessels, thrombosis of dilated vessels, and large grape-like vesicles within the placenta.^[1]^ So far, the pathology is not completely clear. Arizawa M et al supposed VEGF-D (Xp22.31) and IGF-2 (11p15.5) are associated with PMD.^[2]^ Clinically, PMD is often misdiagnosed as a partial hydatidiform mole or complete mole with coexisting fetus. Molar pregnancies belong to a group of diseases classified as gestational trophoblastic diseases, which result from altered fertilization. Partial molar pregnancy with a live fetus is a very rare condition, occurring in 0.005 to 0.01% of all pregnancies. Molar pregnancies belong to a group of diseases classified as gestational trophoblastic diseases, which result from altered fertilization ^[3–4]^. Unlike hydatidiform mole, PMD has no potential for malignancy. Therefore, the treatment and prognosis of these 2 diseases are different, such as choosing to terminate the pregnancy or continue the pregnancy, postpartum observation, or chemotherapy. Here, we presented a case of misdiagnosis of PMD as pregnancy with a hydatidiform mole and reviewed the literature on PMD to investigate its clinical characteristics.

## 2. Case report

### 2.1. The clinical course

**Figure 1.**
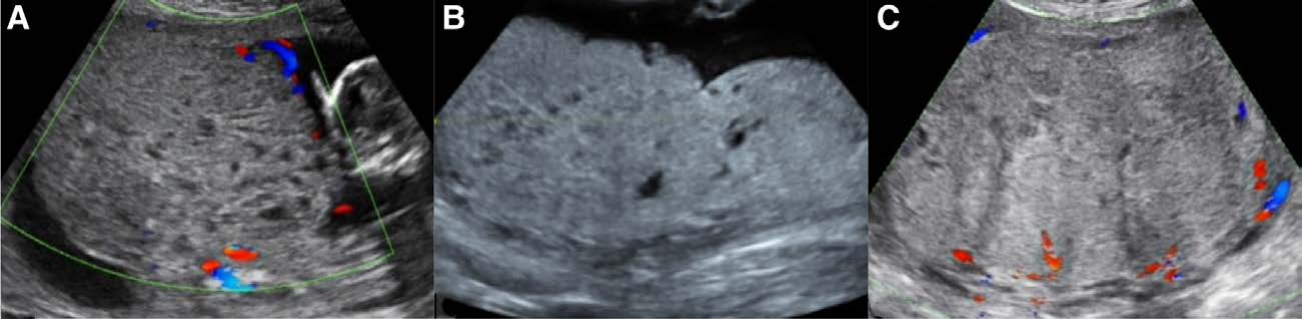
Ultrasound findings of placental mesenchymal dysplasia (PMD). (A) Sonographic image at 15 + 5 weeks’ gestation showing 106 × 68 mm^2^ mixed echo above placenta, partial anechoic area inside, honeycomb like. (B) Sonographic image at 25 + 5 weeks’ gestation showing 142 × 101 mm^2^ mixed echo above placenta, partial anechoic area inside, honeycomb like. (C) Sonographic image at 30 weeks’ gestation showing 144 × 130 × 89 mm^3^ mixed echo above placenta, partial anechoic area in the inner part and point strip blood flow in the inner part.

A 28-year-old female, gravida 2, para 1, who became pregnant through natural fertilization, was referred to our maternal and child health hospital at 15 weeks + 2 days of pregnancy due to ultrasonic diagnosis of partial hydatidiform mole (Fig. 1A), with no obvious abnormality in the fetus. Serologic Down screening at 18 weeks of pregnancy suggested a high risk of neural tube defects (NTD). Laboratory data revealed that the patient had an elevated alpha-fetoprotein (AFP) (multiples of the median, 7.85), and serum human chorionic gonadotropin (HCG) was consistent with gestational weeks. Analysis of chromosome karyotype + mononucleotide-based gene microarray by amniocentesis at the 19th week of gestation showed that fetal amniocentesis chromosome 46, XN, high-resolution chromosome microarray analysis of Affymetrix CytoScan 750K Array revealed a 210 kb fragment deletion in chromosome 2p16.3 containing NRXN1, an OMIM gene, the deleted fragment was derived from a mother with a normal phenotype. The pregnancy was continued following the choice of the patient. At 25 weeks + 5 days and 30 weeks of the pregnancy, the doppler ultrasound showed that 144 × 130 × 89 mm^3^ and 158 × 167 × 89 mm^3^ mixed echo was seen above the placenta, respectively, with honeycomb-shaped anechoic regions in it (Fig. 1B and C). The level of HCG was in the normal range. The pregnant woman felt a headache at 36 weeks + 2 days of the pregnancy. Taking into account the possibility of invasive hydatidiform moles, the pregnant woman was subjected to CT scans of the skull and lungs, and the results were normal. Because of “Scar uterus” and “Pregnancy with hydatidiform mole,” a 2490 g female infant was delivered by cesarean section at 36 weeks + 5 days of gestation with an Apgar score of 9/9. The maternal HCG level decreased to the normal range after 10 days of delivery, and the infant was not found abnormal after 3 months of follow-up. Informed consent was obtained from the parents, and the study was reviewed by the Prenatal Diagnosis Ethics Committee of Jiaxing Maternal and Child Health Care Hospital.

### 2.2. Placental pathology

**Figure 2.**
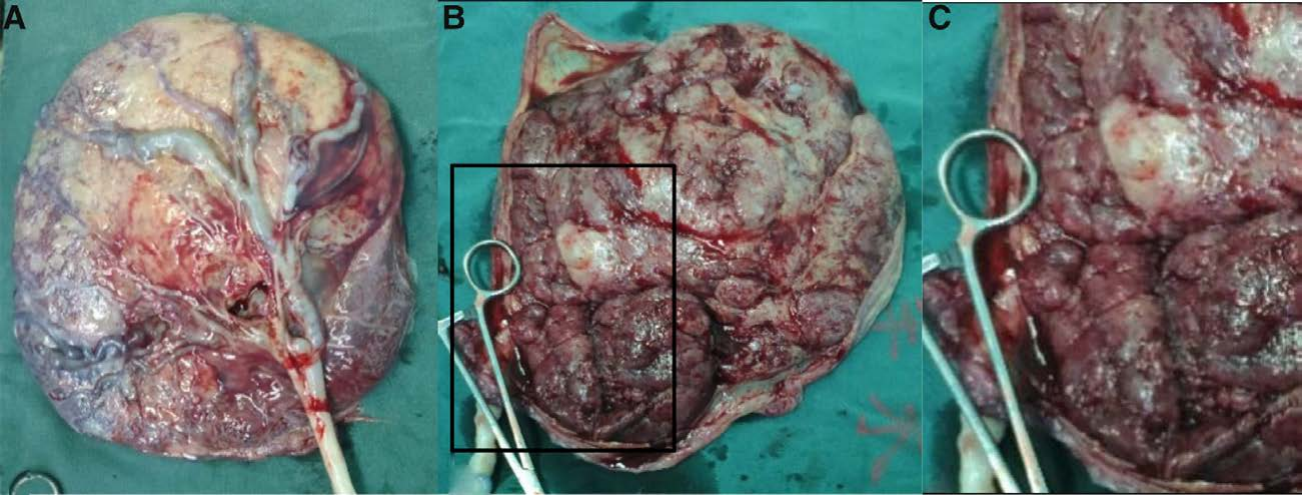
Macroscopic findings of the placenta. (A) Placental fetal surface. (B) Placental maternal surface. It showed multiple cystic lesions, accounting for approximately 30% of the entire placenta. (C) The enlarged view of the cystic lesions.

**Figure 3.**
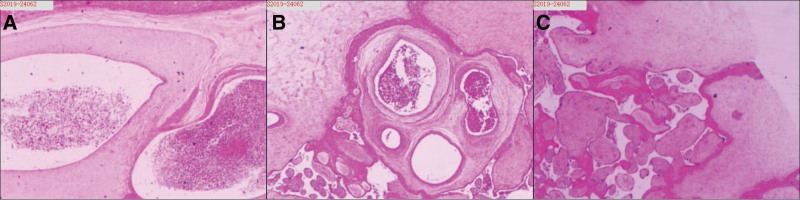
Microscopic findings of the placenta using HE staining. (A)Placental mesenchymal dysplasia shows dilated and congested blood vessels withedema-like changes in the wall. (B) Placental mesenchymal dysplasia showing loose and edematous chorionic plate, cellulose deposition under the plate and part of the chorion, interstitial large blood vessels congestion, and expansion. (C) Placental mesenchymal dysplasia showing partial villi degenerated, cellulose deposited under the chorionic plate and around the villi, interstitial fissure widened, and local hemorrhage occurred. (magnification: 10X).

The size of the placenta was 21 cm × 17 cm × 7 cm (530 g), with multiple cystic lesions about 0.3 to 1.0 cm in size, accounting for approximately 30% of the entire placenta (Fig. 2). The postoperative pathology report revealed part of the villi are mature, most of the placenta are thickened, villous interstitial fibrosis with white infarction and calcification, bleeding and thrombosis was seen in local blood vessels, and the fetal membrane tissue is normal (Fig. 3). The postoperative diagnosis was PMD.

## 3. Literature review

### 3.1. Material and methods

#### 1.3.1. Sources and screening of the literature.

The literature was retrieved from China National Knowledge Infrastructure (201001-202003) established by Tsinghua University (https://www.cnki.net/) and WANFANG DATA (http://www.wanfangdata.com.cn/index.html), with PMD as the indicator from 2010 to 2020. After reviewing the full text, the literature review and duplicate case reports were excluded. Finally, a total of 12 articles were screened.^[3–15]7^ Including this example, 20 pathologically confirmed Chinese cases of PMD were obtained.

## 4. Result

The clinical characteristics of 20 cases of PMD are shown in Table 1. The median maternal age was 27 years (range 23–42 years). Intrauterine death occurred in 7 cases; 1 case of termination of pregnancy due to high risk of NTD; fetal growth restriction occurred in 4 cases; premature delivery occurred in 5 cases (3 fetal distress, 1 premature rupture of membranes, 1 invasive hydatidiform mole brain metastases are suspected); including this case, there are 3 cases of normal fetal growth and development. There were 12 live births and 8 stillbirths, including 1 male fetus, 15 female fetuses, and 4 unreported genders. 1 case with hypoplasia of the left kidney and 1 case with NTD (Table 1).

**Table 1:** Clinical characteristics of cases of placental mesenchymal dysplasia reported in China (201001-202003).

Case	Maternal age	G/ P	Prenatal ultrasound (placenta)	Down screening	Laboratory examination	Pregnancy complication	Delivery [type, cause]	Birth outcome	Adverse neonatal outcomes	Quality of the placenta
1 (CYY)	25	G2/P0	Multiple anechoic areas	High risk of NTD	Fetal karyotype: 46, XX	Postpartum hemorrhage (total hemorrhage 1100 mL)	32 + 1 WG: EmCS; multiple placental cysts with hemorrhage	FGR; female 1580 g; Apgar: 7–8/1–5 min	NICU during 10 days	500 g
2*	28	G2/P1	Honeycomb-shaped heterogeneous echo, considered to be a partial hydatidiform mole	High risk of NTD	Fetal karyotype: 46, XX; serum HCG: within the normal range; AFP:7.85 MOM		36 + 2 WG:EmCS; scar uterus; invasive hydatidiform mole brain metastases considered	Female; 2490 g; Apgar: 9/9/9		
3 (KYQ)	36	G2/P1	Multiple small anechoic areas	High risk of trisomy 21 syndrome		Intrauterine infection	39 WG: EmCS; Scar uterus	FGR; female 1790; Apgar: 9/9	Neonatal anemia, jaundice, hypoproteinemia	590 g
4 (KYQ)	31	G3/P1	Markedly thickened, honeycomb-shaped heterogeneous echo				37 WG: EmCS; scar uterus	FGR; female 2370 g; Apgar: 9/9		579 g
5 (KYQ)	33	G4/P1		Low risk of trisomy 21 syndrome			38 WG: EmCS; scar uterus	Female; 3150 g		510 g
6 (ZY)	24	G3/P1	Part of the cellular structure, considered to be a disease of gestational trophoblastic cells		Serum HCG: within the normal range		15 WG: induction for IUD	Female; stillbirth		
7 (ZY)	23	G1/P0	65% cellular heterogeneous echo, suspected pregnancy trophoblast disease		Serum HCG (mIU/mL): within the normal range		16 WG: induction for IUD	Male; stillbirth		
8 (ZY)	36	G3/P1	Heterogenous echo of placental honeycomb (no change in maximum size)		Serum HCG (mIU/mL): within the normal range		38 WG: the mode of delivery is unknown	Female; Apgar: 10/10		
9 (ZY)	24	G2/P1	The placenta is polycystic near the umbilical cord				30 WG: female, IUD	Female; stillbirth		
10 (ZY)	40	G3/P2	The placenta is polycystic, not excluding trophoblast diseases				15 WG: induction for IUD	Female; stillbirth		
11 (WL)	23		Honeycombed change in the placenta				39 WG: SVD	Female; 2600 g; Apgar: 8/10		
12 (WYN)	27						35 WG: EmCS; conscious fetal movement disappeared, fetal distress was diagnosed	Female; 2000–2499 g	Neonatal asphyxia (severe), followed by death; left kidney dysplasia	535 g
13 (ZYP)	23		A honeycomb mass of 8.8 cm × 4.2 cm × 7.6 cm with an unclear boundary and suspicious partial hydatidiform mole	High risk of NTD	Serum HCG: within the normal range; AFP: 6.22 MOM		35 WG: SVD; premature rupture of membranes	FGR; female 1800 g; Apgar: 10/10/10		760 g
14 (TT)	32		The thickness is about 2.8 cm, scattered in a number of cystic dark areas of different sizes, considering the possibility of partial hydatidiform mole		Serum HCG: >200,000 (mIU/mL)		11 WG: induction for IUD	Stillbirth		
15 (LJY)	27	G3/P0	Multiple anechoic vesicles	NTD(+)			29 + 5 WG: TOP	Stillbirth		
16 (CAW)	23		A mixed mass of 8.6 cm*5.4 cm in size can be seen in the umbilical cord, and a liquid dark area can be seen in it.			Pregnancy with mild anemia	30 + 6 WG: EmCS; fetal distress	1890 g; Apgar: 8/10		570 g
17 (CYH)	30	G1/P0	The local echo is decreased, and the interior is slightly changed like a grid, with a range of 69 mm × 60 mm × 33 mm	Normal			40 WG: SVD; normal fetal condition	Female		Enlarged placenta (no numbers)
18 (CAW) Pathology	24						30 + 6 WG: induction for IUD	Female; 1160 g, stillbirth		490 g
19 (HLJ)	42					Raised blood pressure for half a month (severe preeclampsia)	31 WG: induction for IUD	Stillbirth		550 g
20 (SSL)	23	G1/P0	Multiple anechoic areas, larger areas of about 25 mm × 24 mm			Severe preeclampsia	30 + 1 WG: EmCS; fetal distress	Female; 1250 g; Apgar: 6/9		980 g

AFP = alpha-fetoprotein, EmCS = emergency cesarean, FGR = fetal growth restriction, G/ P = Gravida/ Parity, HCG = human chorionic gonadotropin, IUD = intrauterine death, NICU = neonatal intensive care unit, NTD = neural tube defects, SVD = spontaneous vaginal delivery, TOP = termination of pregnancy, WG = weeks gestation.

Five pregnant women had complications during pregnancy, including 1 postpartum hemorrhage, 1 intrauterine infection, 1 pregnancy with mild anemia, and 2 severe preeclampsia. The results of HCG in serum were recorded in 6 of the 20 cases, all of which were within the normal range except for a slight increase in 1 case; two cases were detected by fluorescence in situ hybridization of chorionic villi cells, and the karyotypes of all fetuses were 46, XX; 7 cases have reported the results of Down screening, 4 cases were at risk of NTD, 2 cases were at risk of Down syndrome, and 1 case had normal results; Placental mass was reported in 10 cases, and the mean placental mass was 606 g.

## 5. Discussion

PMD is a rare clinical syndrome and was first termed by Moscoso et al in 1991,^[16]8^ and Arizawa M et al reported that the incidents of PMD are 0.02% in their hospital.^[2]^ According to a literature search, only 20 cases of PMD have been reported in China in the last century, and it is clear that many cases have been misdiagnosed or missed. The reasons may be as follows. Many pregnant women terminate their pregnancy after being misdiagnosed as a hydatidiform mole. Meanwhile, according to the reported literature (19 PMD cases), PMD can be associated with preterm labor (7/19 or 33%), intrauterine growth restriction (IUGR; 7/19 or 33%), intrauterine fetal death (IUFD; 3/19 or 13%), normal neonatal (2/19 or 9%) or genetic abnormalities such as Beckwith–Wiedemann syndrome.^[17]9^ There is a recent paper showing a key contribution of mesenchymal stromal cells to the altered trophoblast cell cycle regulation typical of preeclampsia with maternal-fetal compromise.^[20]^ Therefore, many pregnant women choose to give birth in advance or induced labor before full term. In addition, even after obtaining a relatively good maternal and infant outcome, due to our inadequate understanding of PMD, no further pathological examination of the placenta was carried out.

Most PMD cases are found abnormal during a routine prenatal ultrasound examination. In this case and 19 cases of PMD reported in the literature, 16 cases were found abnormal by prenatal ultrasound, including multiple anechoic areas and thickened placenta. These manifestations can also be seen in pregnancy trophoblast diseases, especially in partial hydatidiform mole. In the cases we collected, 6 cases, including the present, were diagnosed as a gestational trophoblastic disease by ultrasound. However, we can identify these 2 from the following findings. The cystic part of PMD can show blood flow, while a hydatidiform mole without a living embryo usually has no blood flow; thus, PMD sonograms show stained glass signs that the hydatidiform mole does not have.^[21]^ Apart from this, although PMD may present with elevated HCG levels, the HCG levels in PMD cases are mostly in the relatively normal range, consistent with gestational age.^[17,18]22^ HCG levels were recorded in 5 of the 20 cases, all of which were within the normal range except for 1 case of elevation. Moreover, PMD is often accompanied by an increase in AFP, which may be related to an increase in fetal-maternal surface exchange area.^[16]8^ Meanwhile, elevated AFP levels can lead to fetal misdiagnosis of NTD. Of the cases we collected, 4 were diagnosed as having a high risk of NTD during prenatal screening, and pregnant women were most likely to abandon their fetuses.

PMD has some other clinical features as follows. In our study, 15 of 16 cases are female, consistent with the reported significant female advantage in PMD cases.^[2]^ Some scholars have used androgenetic/biparental mosaicism to explain this phenomenon; the replication results of genomic abnormalities will form 46, YY, and 46, XX cells simultaneously, but the androgen 46, YY cell lines cannot exist.^[23]^ In addition, abnormalities of the vascular endothelial growth factor on the X chromosome can lead to vascular malformations in PMD and female fetuses in PMD.^[2,19]4^ In addition, PMD is often associated with pregnancy-induced hypertension, but whether there is a relationship between them needs further study.

This study has some limitations that are worth noting, such as a small sample size and incomplete clinical data of some patients. In the future, we need to accumulate more cases and improve the data quality.

In conclusion, PMD is a rare clinical disease easily confused with gestational trophoblastic disease. Our study urged that PMD exhibits some unique clinical, laboratory, and imaging features suggesting the histological features of the PMD diagnosis. It is hoped that our study can help sonographers and obstetricians diagnose placental dysplasia correctly, to avoid unnecessary pregnancy termination caused by misdiagnosis.

## Author contributions

**Conceptualization:** Shengfeng Xu.

**Data curation:** Xiaoying Jin.

**Formal analysis:** Jiarui Li.

**Funding acquisition:** Ping Tang.

**Investigation:** Xiaoying Jin.

**Methodology:** Jiarui Li, Yuan Li.

**Resources:** Ping Tang.

**Validation:** Liyan Zhang.

**Writing – original draft:** Xiaoying Jin.

**Writing – review & editing:** Shengfeng Xu.
